# Comparison of SYBR green I real-time RT-PCR with conventional agarose gel-based RT-PCR for the diagnosis of infectious bronchitis virus infection in chickens in Morocco

**DOI:** 10.1186/s13104-016-2037-z

**Published:** 2016-04-22

**Authors:** Siham Fellahi, Mehdi El Harrak, Jens H. Kuhn, Ghizlane Sebbar, El Arbi Bouaiti, Khadija Khataby, Ouafae Fassi Fihri, Mohammed El Houadfi, My Mustapha Ennaji

**Affiliations:** Unit of Avian Pathology, Agronomic and Veterinary Institute Hassan II, B.P. 6202, Rabat, Morocco; Laboratory of Virology, Microbiology and Quality/Ecotoxicology & Biodiversity, Faculty of Sciences and Techniques-University Hassan II Mohammedia, PO Box 146, Quartier Yasmina-Mohammedia, 20650 Casablanca, Morocco; Laboratory of Molecular Biology-Society of Biological Products and Veterinary Pharmaceuticals (Biopharma), B.P. 4569, Km 2, Route de Casa, Rabat, Morocco; Integrated Research Facility at Fort Detrick, National Institute of Allergy and Infectious Diseases, National Institutes of Health, B-8200 Research Plaza, Fort Detrick, Frederick, MD 21702 USA; Laboratory of Epidemiology and Clinical Research. Faculty of Medicine, B.P. 1014, 4, Avenue Ibn Battouta, Rabat, Morocco

**Keywords:** Coronavirus, Gammacoronavirus, IBV, Infectious bronchitis virus, SYBR green, Real time RT-PCR, RT-PCR

## Abstract

**Background:**

A rapid, sensitive, and specific molecular method for the diagnosis of infectious bronchitis virus (IBV) infection is important in curbing infectious bronchitis outbreaks in Morocco and other countries.

**Methods:**

In this study, an easy-to-perform SYBR green I real-time reverse transcriptase polymerase chain reaction (RT-PCR) targeting the nucleocapsid gene of IBV was developed and compared with conventional agarose gel-based RT-PCR for the detection of IBV infection.

**Results:**

We found that the SYBR green I real-time RT-PCR was at least 10 times more sensitive than the agarose gel electrophoresis detection method. The assay exhibited high specificity for IBV infection. All negative controls, such as Newcastle disease virus, infectious bursal disease virus, and avian influenza virus, were not detected.

**Conclusion:**

The SYBR green I real-time RT-PCR test described herein can be used to rapidly distinguish IBV from other respiratory pathogens, which is important for diagnosis and control of infectious bronchitis outbreaks in Morocco. The test is a valuable and useful method as a routine assay for diagnosis of clinical IBV infection in commercial chickens.

## Background

Infectious bronchitis (IB), an acute, highly contagious viral upper respiratory disease of chickens, is one of the most economically significant diseases hampering the intensive poultry industry worldwide. IB affects chickens of all ages, causing respiratory, reproductive, and renal manifestations [1]. Although control of IBV infection is primarily achieved through live attenuated vaccines, the infection is difficult to contain because immunization with different serotypes of the virus do not necessarily cross-protect against other serotypes [2]. Within an infected poultry flock, quick and accurate detection of the presence of the virus is imperative to properly vaccinate uninfected flocks. In addition, rapid differentiation of IBV infection from other upper respiratory tract diseases (e.g., avian influenza, Newcastle disease, infectious laryngotracheitis, avian mycoplasmosis) is important so that appropriate measures can be taken in a timely manner [3].

IB is a disease that negatively impacts the poultry industry of developing countries. For instance, in Morocco, IB continues to be an uncontrolled problem [4–6] due to the lack of in-country diagnostic capabilities that can be performed quickly and interpreted easily by local staff in potentially underequipped or otherwise challenging environments.

The causative agent of IB, infectious bronchitis virus (IBV), is a member of the species *Avian coronavirus*, genus *Gammacoronavirus*, family *Coronaviridae* [7]. IBV is an enveloped, positive-sense, single-stranded RNA virus (genome length = 27.6 kb) [8], expressing three major structural proteins: the nucleocapsid protein (N) surrounding the viral RNA, the membrane glycoprotein (M), and the spike glycoprotein (S) located on the surface of the viral envelope. The S protein contains two post-translational subunits, S1 and S2 [9].

Current diagnostic assays for IBV include virus isolation in embryonated eggs, tracheal organ culture, cell culture immunoassays, and molecular assays that detect viral RNA [10]. Virus isolation has been considered to be the reference standard. However, such isolations are expensive and time consuming because several passages may be required to detect the virus. Immunoassays use IBV-specific monoclonal antibodies to detect the virus in direct or indirect fluorescent antibody and enzyme-linked immunosorbent assay (ELISA) formats. Although faster and simpler than virus isolation, immunoassays tend to lack specificity and sensitivity. None of these immunoassays detect all strains or types of IBV [11–13]. Molecular assays, such as reverse transcriptase-polymerase chain reaction (RT-PCR), for the detection of IBV are commonly used because highly specific and sensitive results can be obtained in a timely manner. Molecular assays detect viral RNA directly from a clinical sample or from virus isolated in a laboratory host system. When RT-PCR is used to amplify the spike glycoprotein (S) of IBV, the assay can be coupled with restriction fragment length polymorphism analysis or nucleic acid sequencing to identify serotypes of the virus [2, 10, 12–16]. More recently, many fluorescent probe-based real-time RT-PCR assays have been developed to detect IBV strains [2, 3, 9, 10, 15, 17]. Real-time TaqMan RT-PCR assays have been developed that amplify a fragment of the 5′ untranslated region of the IBV genome to detect turkey coronaviruses and IBV [17] or that target the N gene for IBV detection [9].

Unfortunately, performing most of these assays requires highly trained staff, a sophisticated infrastructure, or considerable monetary funds, and are therefore not necessarily viable options for developing countries such as Morocco. SYBR green I-based RT-PCR assays have proven to be among the most effective tools in the rapid and differential detection of a variety of viral diseases such as avian influenza, Newcastle disease, and IB. These inexpensive and easily performed assays are important to rapidly identify the causative agent of any upper respiratory disease or changes in egg shell quality and egg production in chickens [17, 18].

However, an assay employing real-time RT-PCR with SYBR green I dye to target the N gene of IBV is lacking. Here we report the development of a real-time RT-PCR assay with SYBR green I dye for rapid detection of IBV viral RNA directly from Moroccan clinical samples. We also compared the assay with conventional RT-PCR and agarose gel electrophoresis to detect IBV PCR-amplified products.

## Results

### Reproducibility

#### SYBR green I real-time RT-PCR

**Fig. 1:**
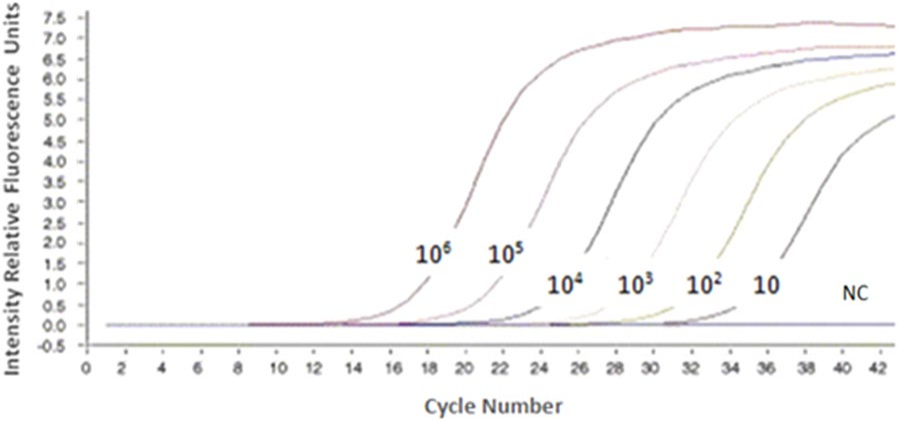
Real-time reverse transcriptase polymerase chain reaction for detection of infectious bronchitis virus. cDNA derived from IBV Beaudette strain genomic RNA was diluted serially from 10^6^ to 10^1^ copies/μl and detected by real-time PCR. The *x-axis* indicates the PCR cycle number, whereas the *y-axis* indicates the fluorescence intensity over the background. *NC* negative control, *PCR* polymerase chain reaction

The cDNA IBV Beaudette strain (infectious bronchitis virus/G.gallus-wt/FRA/1995/Beaudette) was used to extract the standard control genomic RNA that then served as the source of cDNA template. Concentrations of the cDNA standard ranging from 10–10^6^ copies/µl were tested repeatedly (Fig. 1) by SYBR green-I-based real-time RT PCR. The results are reproducible through 10 cDNA copies/μl.

#### Conventional gel-based RT-PCR

**Fig. 2:**
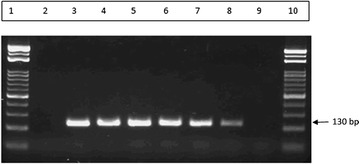
Agarose gel electrophoresis of conventional RT-PCR amplified products from infectious bronchitis virus N gene. Electrophoresis of PCR amplification after serial dilutions of the IBV Beaudette strain in a 1.5 % agarose gel stained with ethidium bromide. *Lanes 1* and *10* 50-bp DNA ladder; *lane 2* negative control; *lane 3* 10^8^ copies/μl; *lane 4* 10^7^ copies/μl; *lane 5* 10^6^ copies/μl; *lane 6* 10^5^ copies/μl; *lane 7* 10^4^ copies/μl; *lane 8* 10^3^ copies/μl; *lane 9* 10^2^ copies/μl. *bp* base pairs, *IBV* infectious bronchitis virus, *RT*-*PCR* reverse transcriptase-polymerase chain reaction

The PCR targeted a sequence corresponding to a region of the IBV N protein. The obtained PCR products of 130 bp in length were separated on a 1.5 % agarose gel stained with ethidium bromide (Fig. 2). PCR products were detectable at a dilution of 100 cDNA copies/µl.

### Specificity of SYBR green I real-time RT-PCR

**Fig. 3:**
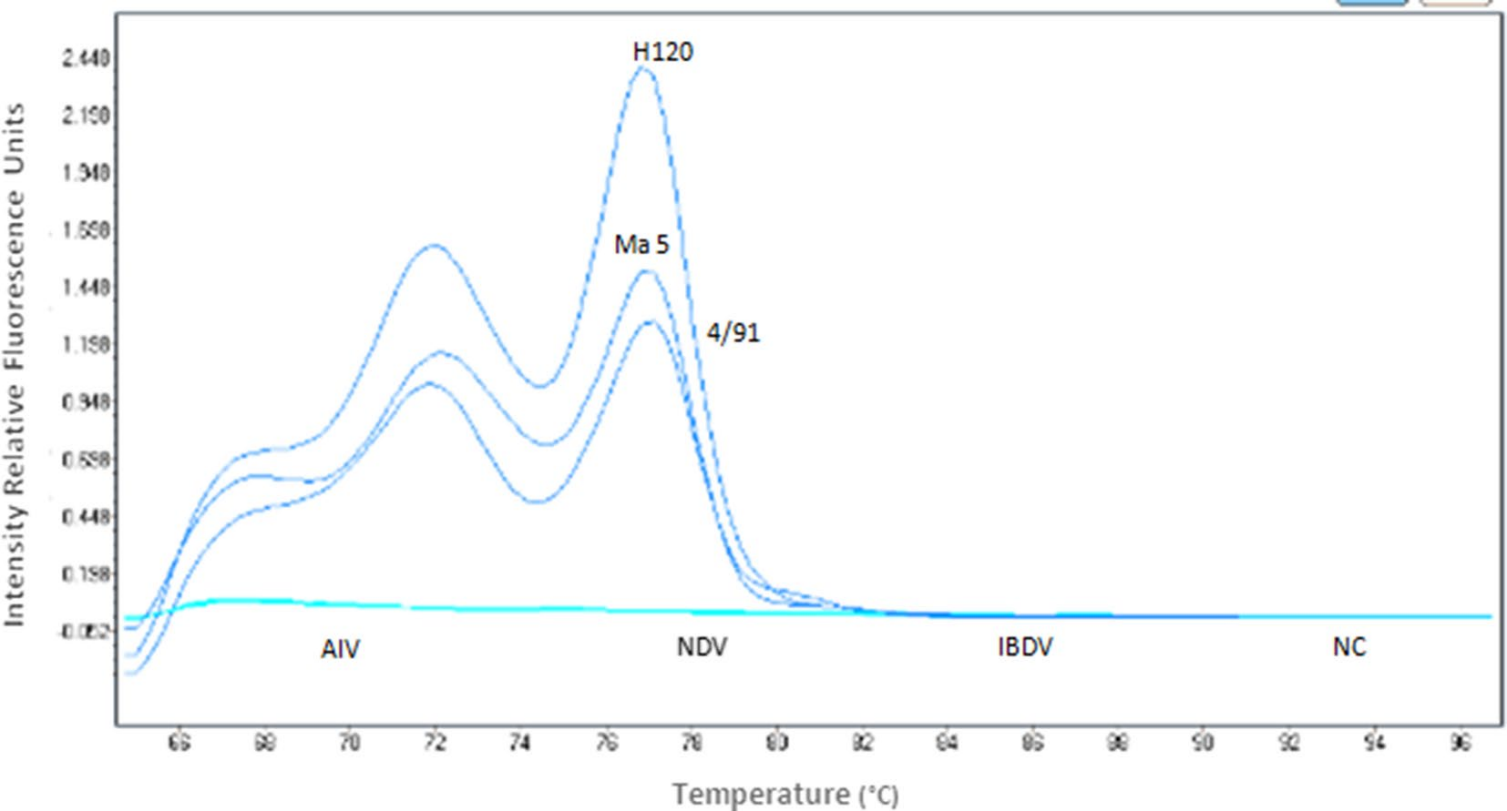
Specificity of IBV N gene-based SYBR green I real-time RT-PCR. Amplification plot representing IBV (H120, Ma5, 4/91), FLUAV, IBDV, NDV, and negative control (NC). The *blue curve* shows the amplification plots of the IBV-positive samples while the *turquoise curve* shows the plots of the IBV-negative strains. *FLUAV* avian influenza virus A, *IBV* infectious bronchitis virus, *IBDV* infectious bursal disease virus, *NDV* newcastle disease virus

Melting peaks analysis on the PCR products of H120, MA5, and 4/91 vaccine strains and tenfold serially diluted cDNA (data not shown) did not indicate primer-dimers or nonspecific products. Specific amplification of the IBV target sequence was identified by the generation of a melt peak at 68 ± 0.20 °C. The specificity of the SYBR green-I real-time RT-PCR was 100 % since detectable fluorescent signals were not observed with the negative control (NC), avian influenza virus (FLUAV), infectious bursal disease virus (IBDV), and Newcastle Disease virus (NDV) nucleic acids. Only the IBV vaccine strain genetic material was detected (Fig. 3).

### Sensitivity

#### SYBR green I real-time RT-PCR

**Fig. 4:**
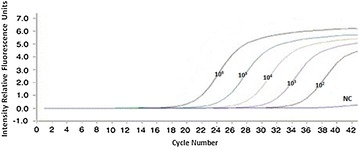
SYBR green I real-time RT-PCR amplification plot derived from cDNA of IBV H120 vaccine strain. *IBV* infectious bronchitis virus, *RT*-*PCR* reverse transcriptase-polymerase chain reaction

To determine the sensitivity endpoint of the assay, serial dilutions of cDNA at concentrations ranging from 1.25 to 100 copies/µl were analyzed. The real-time RT-PCR fluorescence curve derived from serially diluted standard concentrations (cDNA of H120 vaccine strain) indicates a high sensitivity of detection down to 100 copies/µl of cDNA (Fig. 4).

**Fig. 5:**
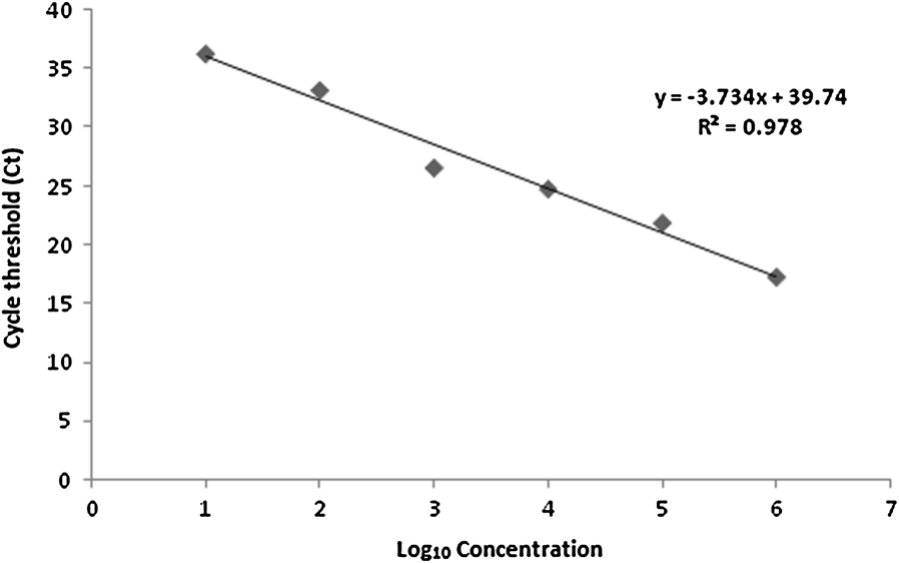
Simple linear regression line of Ct values vs. log_10_ of tenfold serial dilutions (10^6^–10 copies/µl) of standard RNA genome of cDNA of H120 vaccine strain. *Ct* cycle threshold

To determine the linearity of the reaction and the RT-PCR efficiency, the cycle threshold (Ct) values of individual dilutions were analyzed. The assay has a dynamic detection range that spanned 10^2^–10^6^ cDNA copies/µl. Except for the undiluted cDNA, a strong inverse linear relationship was observed between the amount of input cDNA and the Ct values over six log_10_ dilutions (Table 1), as indicated by a simple linear regression plot of the two variables (Fig. 5).

**Table 1:** Sensitivity and specificity of Sybr green I real-time RT-PCR in detection of cDNA from the IBV H120 vaccine strain compared to conventional RT-PCR agarose gel electrophoresis

cDNA concentration (log_10_ copies/µl)	Detection method						
	SYBR green I real-time RT-PCR						Conventional RT-PCR
	Ct value	Sensitivity		Specificity		P/N	P/N
		%	CI	%	CI		
10^1^	36.31	0	0–69.5	100	19.3–100	−	−
10^2^	33.19	33.33	5.5–88.4	100	19.3–100	+	−
10^3^	26.59	66.67	11.6–94.5	100	19.3–100	+	+
10^4^	24.79	100	30.5–100	100	19.3–100	+	+
10^5^	21.90	100	30.5–100	50	8.2–91.8	+	+
10^6^	17.29	100	30.5–100	0	0–80.7	+	+

*CI* confidence interval, *Ct* average cycle threshold, *RT*-*PCR* reverse transcriptase-polymerase chain reaction, + positive (P), − negative (N)

#### Conventional RT-PCR

**Fig. 6:**
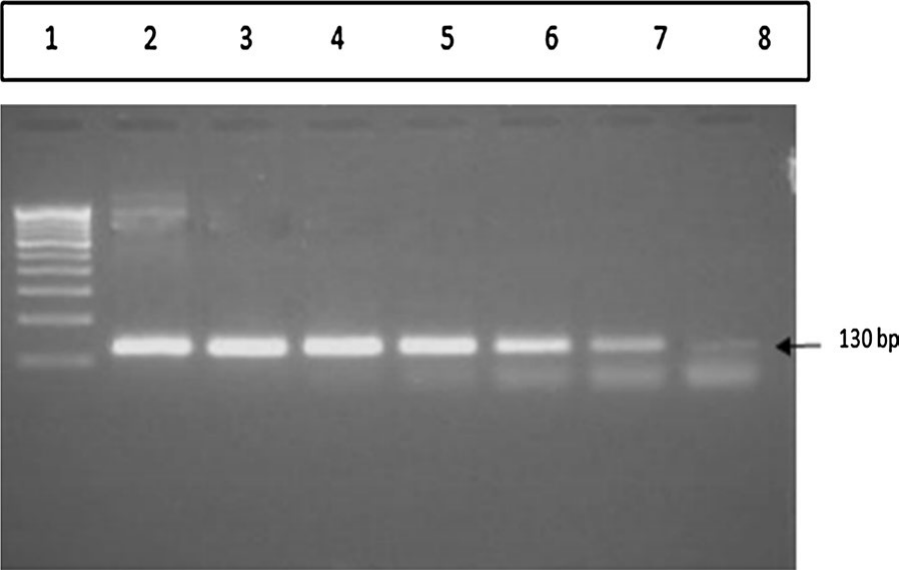
Agarose gel electrophoresis of conventional RT-PCR products from the N gene of IBV H120 strain. Amplified products were separated by electrophoresis on a 1.5 % agarose gel and stained with ethidium bromide. *Lane 1* 100 bp DNA ladder; *lane 2* 10^7^ copies/μl; *lane 3* 10^6^ copies/μl; *lane 4* 10^5^ copies/μl; *lane 5* 10^4^ copies/μl; *lane 6* 10^3^ copies/μl; *lane 7* 10^2^ copies/μl; *lane 8* 10 copies/μl. *IBV* infectious bronchitis virus, *RT*-*PCR* reverse transcriptase-polymerase chain reaction

Results from a conventional RT-PCR using the IBV N gene confirmed that the obtained RT-PCR products were 130 bp in length, and the PCR products were detectable at a concentration of 100 copies/µl (Fig. 6).

### Comparison of sensitivity and specificity of the SYBR green I real-time RT-PCR assay with conventional RT-PCR

**Fig. 7:**
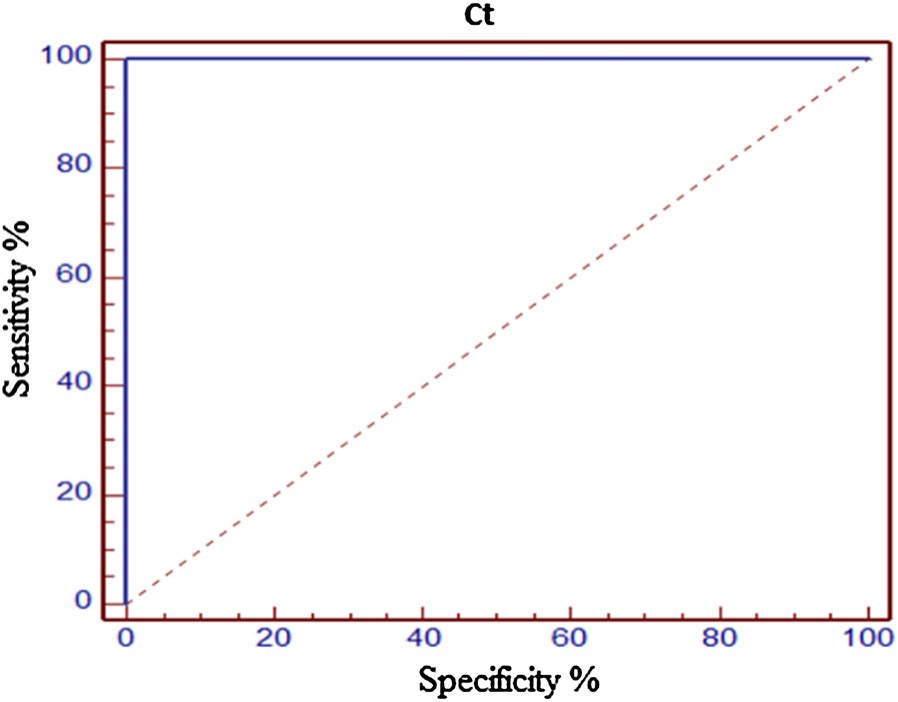
Receiver operating characteristic curve analysis of Ct values obtained from SYBR green-I-based real-time RT-PCR versus conventional RT-PCR. *RT*-*PCR* reverse transcriptase-polymerase chain reaction

The lowest dilution of cDNA that SYBR green I real-time RT-PCR assay was able to detect was an order of magnitude higher than conventional RT-PCR assay (Table 1). Receiver operating characteristic curve analysis of Ct values obtained from the SYBR green I real-time RT-PCR assay indicates the linearity of the reaction and the assay’s efficiency (Fig. 7). The assay has a maximum sensitivity and specificity over a detection range that spanned 10^4^ cDNA gene copies/µl (Table 1).

### Detection of IBV in tracheal swabs from clinical samples

**Fig. 8:**
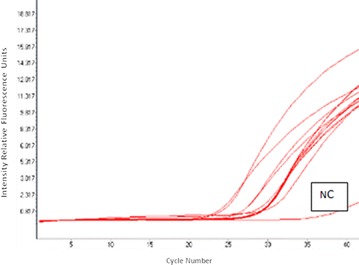
Virus detection in tracheal swabs with SYBR green I real-time RT-PCR. Tracheal swabs (34) were taken from broiler chickens showing clinical signs of infectious bronchitis. A subset of the results from 34 tracheal swabs using SYBR green I real-time RT-PCR are shown. *NC* negative control, *RT*-*PCR* reverse-transcriptase polymerase chain reaction

The optimized methods of SYBR green I real-time RT-PCR and conventional RT-PCR were applied to clinical samples from broiler chickens showing clinical signs of IB, including coughing, sneezing, rales, and nasal discharge. From 34 tracheal swabs tested, all samples and positive controls were positive for IBV by SYBR green I real-time RT-PCR targeting the IBV N gene (data not shown and Fig. 8 for a subset of the total swabs tested). However, only 28 of 34 samples were positive for IBV by conventional RT-PCR (data not shown).

### Intra- and inter-assay variability

The SYBR green I real-time RT-PCR assay demonstrated high repeatability with coefficients of variation within runs (intra-assay variability) and between runs (inter-assay variability) that ranged from 0.6–1.5 and 0.6–1.8 %, respectively (Table 2).

**Table 2:** Intra- and inter-assay reproducibility of SYBR green-I-based real-time RT-PCR

Copy number	Mean crossing point	Intra-assay CV (%)	Inter-assay CV (%)
10^1^	32.90	1.5	1.6
10^2^	28.25	1.2	1.8
10^3^	25.17	0.9	1.4
10^4^	21.84	0.7	1.2
10^5^	18.26	0.6	0.7
10^6^	16.63	0.6	0.6

*CV* coefficient of variation

### One-step RT PCR amplification of the IBV S1 gene and sequence analysis

**Fig. 9:**
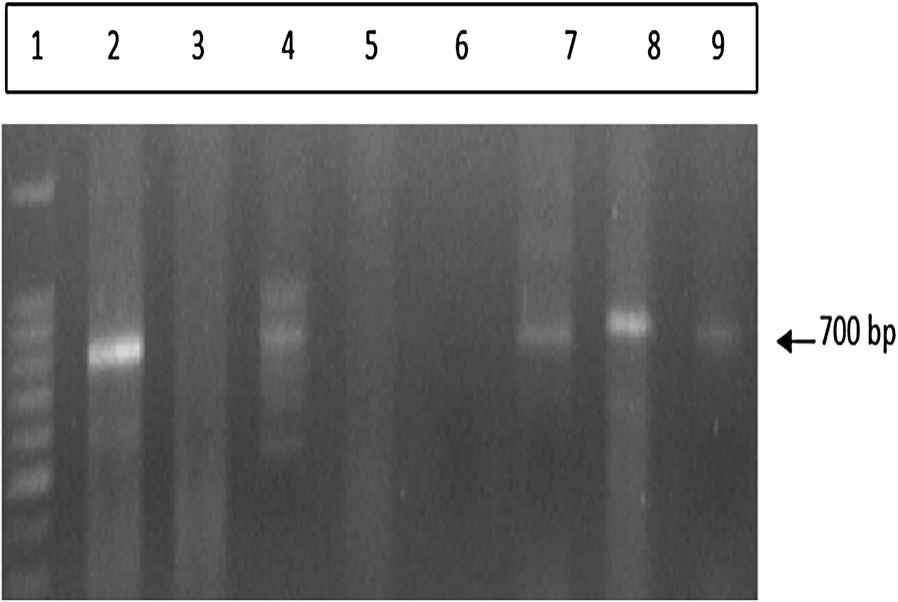
Agarose gel electrophoresis of one-step RT-PCR amplified products of the IBV S1 gene. Commercial IBV vaccine (IBV H120, MA5, and 4/91) and field strains (“Moroccan 30” and “Moroccan 38”) were separated by electrophoresis on a 1.5 % agarose gel stained with ethidium bromide. *Lane 1* 100 bp DNA ladder; *lane 2* Moroccan 30 strain; *lane 3* negative control; *lane 4* Moroccan 38 strain; *lanes 5, 6* negative controls; *lane 7* Ma5 commercial vaccine; *lane 8* H120 commercial vaccine; *lane 9* 4/91 commercial vaccine. Product size, 700 bp. *Bp* base pairs, *IBV* infectious bronchitis virus, *RT*-*PCR* reverse transcriptase-polymerase chain reaction

For further confirmation of our results, we investigated whether tracheal samples that tested IBV-positive using SYBR green I real-time RT-PCR targeting the IBV N gene would also be positive using a one-step RT-PCR targeting the IBV S1 gene. The IBV S1 gene of IBV commercial vaccine strains (H120, Ma5 and 4/91) and field strains “Moroccan 30” and “Moroccan 38” (randomly chosen from the 34 broiler chicken samples) were detected and amplified using a one-step RT-PCR. The obtained RT-PCR product, 700 bp, corresponded to the predicted size (Fig. 9) [19]. The obtained sequences were aligned with those of IBV deposited in GenBank (H120 [M21970]; Ma5 [AY561713]; Italy 02 [AJ457137]; Italy 497/02-1 [DQ901377]; Ark/C6d [EU283056]; QX [AF193423]; strain 04/1991 [AF093794]; and D274 [X15832]), using the BLAST software. The results of sequenced field strains compared to IBV reference strains confirm that the amplified sequences corresponded to the IBV reference strains [GenBank: KJ701019 and KJ701020].

## Discussion

The use of PCR for diagnosis of viral diseases has increased to the point that the assay is now considered the gold standard instead of viral isolation [20]. Real-time PCR has catalyzed wider acceptance of PCR as a diagnostic tool because it is more rapid, sensitive, and reproducible, and the risk of carryover contamination is minimized compared to conventional PCR [21].

Diagnosis of IB is commonly based on virus isolation in embryonated eggs, followed by immunological identification of the isolates. This procedure is time consuming and requires use of specific polyclonal or monoclonal antibodies, which may not be available. Moreover, some isolates could be mixtures of different serotypes of IBV that can confuse interpretation of results. RT-PCR techniques using RNA extracted from allantoic fluid and tracheal swabs from IBV-infected chickens are very efficient in the detection of IBV [22, 23]. However, conventional RT-PCR is time consuming, prone to error, and is less sensitive than real-time RT-PCR [3, 9, 24–26].

With the SYBR green I real-time RT-PCR assay that we developed, we compared the performance of this novel technique to conventional agarose gel-based RT-PCR in detecting specific IBV RT-PCR products. Based on serially diluted cDNA standards, the SYBR green I real-time RT PCR detected down to 100 cDNA copies/µl of the IBV H120 vaccine strain genomic material. The SYBR green I real-time RT PCR assay targeting the highly conserved N gene has a higher sensitivity than conventional RT-PCR in detecting IBV nucleic acids. Targeting the N gene has been postulated to be a better choice to improve the sensitivity of the PCR compared to the S1 gene, as the N protein is abundant in infected cells [9, 27].

The specificity of our test was demonstrated by the absence of positive reactions with genetic material obtained from other RNA viruses, such as FLUAV, IBDV, and NDV (Fig. 3). The optimal melting peak of IBV amplicons strains is 68 ± 0.20 °C. No primer-dimer or nonspecific products were detected. These results are comparable with previously reported SYBR green I real-time RT-PCR assays established for coronaviruses and type-specific IBV multiplex SYBR green I real-time RT-PCR [24, 28].

SYBR green I real-time RT-PCR and conventional RT-PCR were applied to 34 clinical samples obtained from Moroccan broiler chicken flocks showing clinical signs of IB. IBV N gene amplification with SYBR green I real-time RT-PCR detected all the IBV strains assessed, whereas only 28 of 34 clinical swabs tested were positive for IBV by conventional RT-PCR. The increased sensitivity of real-time RT-PCR compared to conventional RT-PCR might be due to detection of the fluorescent signal emitted by specific amplification products [24]. Therefore, the SYBR green I real-time RT-PCR assay developed here using Moroccan field cases could be an important tool for the screening of samples during IB-suspected cases as the assay is rapid, simple, efficient, highly specific, and sensitive.

Recently, real-time RT-PCR assays were described that can detect IBV either solely or in a multiplex set [24, 26, 28, 29]. However, none of these assays utilized the SYBR green I chemistry for specific detection and quantification of IBV targeting the N gene. The main advantages of SYBR green I assays over other real-time PCR detection formats are: (1) SYBR green I is a low-cost fluorochrome; (2) SYBR green I assays are simpler to use, especially in regard to primer design and optimization procedures [25]; and (3) artifacts commonly observed in specific probes, particularly at amplification cycles beyond the 30th round, are minimal and can be ruled out by melting curve analysis. The assay described here is therefore ideal for establishing IB diagnostic capabilities in developing countries such as Morocco.

## Conclusions

In conclusion, we report for the first time a one-step SYBR green I real-time RT-PCR protocol for IBV N gene detection. The assay is a sensitive, simple, efficient, and cost-effective method that can be applied easily for laboratory diagnosis of IBV infections.

## Methods

### Viruses

IBV was propagated in 9–11-day-old embryonated specific pathogen-free chicken eggs as described previously [30]. The allantoic fluid was harvested 48 h post-inoculation and stored at −80 °C until RNA extraction. The different viruses used in this study are listed in Table 3.

**Table 3:** Viruses used in the study

Virus	Strain	Source Type of vaccine
Infectious bronchitis virus (IBV)	H120	Biopharma Massachussetts serotype
	Ma5	MSD Animal Health Nobilis IB Ma5 vaccine
	4/91	MSD Animal Health Nobilis IB 4/91 vaccine
	Beaudette	Ploufragan-Plouzané laboratory Wild attenuated strain
Newcastle disease virus (NDV)	Lasota	Biopharma HB1 type, LaSota strain
Infectious bursal disease virus (IBDV)	Moroccan field strain	Biopharma Vaccine strain
Avian influenza A virus (FLUAV)	H5N1 subtype	Biopharma Inactivated vaccine strain

### Clinical standard samples

To validate the reliability of SYBR green-I-based real-time RT-PCR, the quantified IBV Beaudette strain was used as the standard control, and allantoic fluid with different titers of the virus were obtained.

### Tracheal swabs from IBV-infected chickens

Tracheal samples were obtained from the Avian Unit, Agronomy and Veterinary Institute Hassan II, Rabat, Morocco. These samples were identified during routine diagnostic efforts and stemmed from 34 commercial broiler chickens with clinical signs indicative of IB, including coughing, sneezing, rales, and nasal discharge. All samples had been confirmed to be IBV-positive by virus isolation, but serotypes were not determined. For this study, all samples were tested by SYBR green-I-based real-time RT-PCR and conventional RT-PCR. The swabs were suspended in 300 µl of phosphate-buffered saline, clarified by centrifugation at 1500×*g* at 4 °C for 15 min, and incubated at room temperature for 1 h. The virus-containing supernatants obtained from the tracheal swabs were used for direct extraction of viral RNA.

### RNA extraction

IBV RNA and negative control RNA (NDV [183.6 ng/µl], IBDV [67.5 ng/µl], FLUAV [92.1 ng/µl]) were extracted from 140 µl of supernatant from allantoic fluid using the QIAamp Viral RNA or RNeasy Mini Kits (Qiagen, Valencia, CA, USA) according to the manufacturer’s instructions. Each RNA fraction was eluted in 50 µl of RNase-free water.

### SYBR green-I-based real time RT PCR

SYBR green I-based real time RT-PCR was performed using a primer pair consisting of (a) downstream primer, AIBV-fr, targeting N gene nucleotide positions 811–832 (5’-ATGCTCAACCTTGTCCCTAGCA-3′); and (b) upstream primer, AIBV-as, targeting N gene nucleotide positions 921–941 (5′-TCAA-ACTGCGGATCA-TCACGT-3′, TIB Molbiol, Berlin, DE) as previously described by Meir et al. [9]. To minimize primer-dimer formation, primer set concentration and thermocycling conditions were both optimized (data not shown).

PCRs were performed on a Light Cycler (Roche Diagnostics Ltd. Rotkreuz, CH). Each reaction was carried out using 5 µl of purified RNA and 20 µl of a reaction mixture, containing 0.5 µl of RT (50 U) (Applied Biosystems, Grand Island, NY, USA), 0.5 µl of RNase inhibitor (20 U), 0.5 µl of MgCl_2_ (50 mM), 2 µl of FastStart DNA Master Mix SYBR green I (containing Taq DNA polymerase, SYBR green I, deoxynucleotide triphosphate mix [Life Technologies, Grand Island, NY, USA]), and 0.5 µl of the two primers (final concentration of each primer is 10 µM) and enough nuclease-free water (Promega, Madison, WI, USA) for a final volume of 20 µL for each reaction. The thermal profile consisted of 10 min of reverse transcription at 50 °C, 10 min of hot-start enzyme activation at 95 °C, followed by 45 cycles of PCR at 95 °C for 10 s (denaturation), 58 °C for 20 s (annealing), and 72 °C for 30 s (elongation). To avoid cross contamination and sample carryover, pre- and post-PCR sample processing were performed in separate rooms. Plugged pipette tips were used to transfer all fluids to eliminate aerosols. Amplified cDNA products were detected by melting curve analysis that consisted of 95 °C for 5 s and 65 °C for 60 s, and heated to 97 °C to measure continuous changes in fluorescence of SYBR green I. Following amplification, a melting curve analysis was performed with the Light Cycler instrument’s software (software release 1.5.0, version 1.5.0.39) according to the instructions of the manufacturer. Negative template control (diethyl pyrocarbonate-treated water) and positive template controls (i.e., NDV, IBDV, FLUAV) were included with each PCR run. Each strain used was tested in triplicate.

### Reproducibility of SYBR green-I-based real-time RT-PCR

To evaluate reproducibility of the assay, quantification analysis of IBV cDNA was developed using the 34 clinical standard samples from Moroccan broiler chickens. cDNA of the genomic RNA of IBV Beaudette strain was used as the source of DNA templates. After quantification, IBV Beaudette strain cDNA was serially diluted (10^6^–10 copies/µl) and used as a standard control. Three separate dilution series were assayed in a single run to evaluate intra-assay variations, whereas inter-assay variations were measured by testing each dilution in three separate consecutive runs. The standard deviation (SD) was calculated using Light Cycler Software 480 version 1.5 (Roche Diagnostics Ltd. Rotkreuz, CH). The coefficient of variation (CV) was determined following the formula CV = (SD [Ct-value]/overall mean [Ct-value]) × 100.

### Sensitivity of SYBR green-I-based real-time RT-PCR

The sensitivity of the SYBR green-I-based real-time RT-PCR assay was tested by the limiting dilution assay [31]. Genomic RNA from attenuated Massachusetts serotype H120 vaccine was then diluted to ≈100 (151.6 ng/μl), 20 (35.6 ng/μl), 5 (12.9 ng/μl), and 1.25 (0.7 ng/μl) copies/μl as previously described [32]. The tubes at each concentration were assayed using the forward and reverse primers. Samples with Ct values of less than 32 were considered positive.

### Conventional reverse transcriptase polymerase chain reaction

Conventional RT-PCR was performed in a thermal cycler (Techne, Staffordshire, UK), according to the instructions provided by the manufacturer. The RT-PCR was performed in one step, in a final volume of 20 μl. Briefly, the final volume consisted of 2.5 µl of buffer, 2.5 µl of MgCl_2_ (25 mM), 2.5 µl of deoxyribonucleotide triphosphates (10 mM), 9.7 µl of water, 0.5 µl of RNase inhibitor (20 U), 0.3 µl of reverse transcriptase (50 U), 0.5 µl of Taq polymerase (Gold) (5 U), and 0.75 µl of each of the primers (forward and reverse) (10 µM).

One step, reverse transcription was performed at 48 °C for 30 min and 95 °C for 5 min. cDNA was amplified through a 35-cycle PCR consisting of denaturation at 94 °C for 30 s, annealing at 52 °C for 30 s, and extension at 72 °C for 30 s, with one final extension cycle at 72 °C for 10 min. The amplified PCR products of the N gene were evaluated by 1.5 % agarose gel electrophoresis in Tris–borate–EDTA buffer stained with ethidium bromide (Promega).

### Comparison of sensitivity and specificity of SYBR green I real-time RT-PCR with conventional RT-PCR assay

The analytical sensitivity and specificity of the SYBR green I real-time RT-PCR assay were determined by testing sequential tenfold dilutions of the in vitro-transcribed RNA, which were obtained as previously described, in nuclease-free water (Promega).

### One-step RT-PCR amplification of the IBV S1 gene

PCR amplification of the IBV S gene was carried out using commercial vaccines (H120, Ma5 and 4/91) and field strains, “Moroccan 30” and “Moroccan 38” from chickens with respiratory signs. Each 25 µl of PCR reaction mixture consisted of 2.5 μl of buffer (10X), 2.5 μl of MgCl_2_ (25 mM), 2.5 μl of deoxynucleotide triphosphates (10 mM), 0.75 μl of each primer (10 μM) (CK2 and S15mod) [19], and 9.7 μl of sterilized water. At the end, 0.5 μl of RNAase inhibitor (20 U/μl), 0.3 μl of RT (50 U/μl), 0.5 μl of Taq gold polymerase (ThermoFisher Scientific) (5 U/μl), and 5 µl of IBV RNA were added. This one-step RT-PCR reaction was carried out using Smart Thermal Cycler, (Smart Cycler Cepheid, Sunnyvale, CA, USA) following the protocol of 48 °C for 30 min, 95 °C for 5 min, 40 cycles of 95 °C for 30 s, 52 °C for 30 s, 72 °C for 30 s, and a final extension cycle of 72 °C for 10 min.

### Nucleotide sequencing and sequence analysis

Following amplification, 20 μl of each S1 PCR reaction for “Moroccan 30” and “Moroccan 38” field strains and vaccine strains (H120, Ma5 and 4/91) were subjected to 1.5 % agarose gel electrophoresis (under 70 V for 30 min) and stained with ethidium bromide. The appropriate bands were excised and purified using the Gene Clean Kit (ExoSAP-IT, Affymetrix, Santa Clara, CA, USA) according to the manufacturer’s recommendations. Nucleotide sequences were determined using the same primers (S15mod and CK2) [19] and the BigDye^®^ Terminator v1.1 Cycle Sequencing Kit (Life Technologies, Grand Island, NY, USA). The second purification was performed by Big Dye X terminator Purification Kit.

### Statistical analysis

The significant differences among the mean of the Ct values corresponding to each viral dilution were analyzed by one-way analysis of variance (ANOVA) using Statistical Package for the Social Sciences (SPSS) Version 13 software (IBM, Armonk, NY, USA). A receiver operating characteristic curve statistical test was used to compare the sensitivity and specificity of the SYBR-green-I-based real-time RT-PCR over the conventional RT-PCR. A *p* value of less than 0.05 is considered statistically significant.

## Abbreviations

bp: base pairs
Ct: cycle threshold
ELISA: enzyme-linked immunosorbent assay
FLUAV: avian influenza virus
IB: infectious bronchitis
IBDV: infectious bursal disease virus
IBV: infectious bronchitis virus
M: membrane protein
N: nucleocapsid protein
NC: negative control
NDV: newcastle disease virus
RNA: ribonucleic acid
RT-PCR: reverse transcriptase-polymerase chain reaction
S: spike protein
